# Starch-Based Polysaccharide Systems with Bioactive Substances: Physicochemical and Wettability Characteristics

**DOI:** 10.3390/ijms25094590

**Published:** 2024-04-23

**Authors:** Agnieszka Ewa Wiącek, Anna Furmaniuk

**Affiliations:** Department of Interfacial Phenomena, Faculty of Chemistry, Maria Curie-Skłodowska University, Maria Curie-Skłodowska Sq. 3, 20-031 Lublin, Poland; anna.furmaniuk@poczta.umcs.lublin.pl

**Keywords:** starch, phosphatidylcholine, lysozyme, effective diameter, zeta potential, dynamic light scattering, electrophoresis, wettability, contact angle, hysteresis

## Abstract

Polysaccharide-based systems have very good emulsifying and stabilizing properties, and starch plays a leading role. Their modifications should add new quality features to the product to such an extent that preserves the structure-forming properties of native starch. The aim of this manuscript was to examine the physicochemical characteristics of the combinations of starch with phospholipids or lysozymes and determine the effect of starch modification (surface hydrophobization or biological additives) and preparation temperature (before and after gelatinization). Changes in electrokinetic potential (zeta), effective diameter, and size distribution as a function of time were analyzed using the dynamic light scattering and microelectrophoresis techniques. The wettability of starch-coated glass plates before and after modification was checked by the advancing and receding contact angle measurements, as well as the angle hysteresis, using the settle drop method as a complement to profilometry and FTIR. It can be generalized that starch dispersions are more stable than analogous *n*-alkane/starch emulsions at room and physiological temperatures. On the other hand, the contact angle hysteresis values usually decrease with temperature increase, pointing to a more homogeneous surface, and the hydrophobization effect decreases vs. the thickness of the substrate. Surface hydrophobization of starch carried out using an *n*-alkane film does not change its bulk properties and leads to improvement of its mechanical and functional properties. The obtained specific starch-based hybrid systems, characterized in detail by switchable wettability, give the possibility to determine the energetic state of the starch surface and understand the strength and specificity of interactions with substances of different polarities in biological processes and their applicability for multidirectional use.

## 1. Introduction

Polysaccharides are used in many areas of human life and industries, e.g., in food, pharmaceutical, cosmetics, medicine productions (as dressing materials, medications for use on the skin, for construction of contact lenses, surgical implants, catheters, diaphragms for artificial kidneys), and the production of paints and cleaning agents. One of the most common polysaccharides is starch, the most important in the food industry, which fulfills various functions, e.g., gelling, stabilizing, and clarifying, as well as the inhibition of the crystallization of sucrose as fat substitute or emulsifier [1,2,3,4,5,6,7,8,9,10].

Thanks to the possibility of forming specific ordered spatial structures by appropriate polymer chains, polysaccharides, including starches of various origins, have the ability to form hydrocolloids, which are associated with the presence of functional groups capable of interacting with other chains or molecules. Such interactions through hydrogen and/or coordination bonds can occur between fragments of the same chain as well as between two or more different chains. Starch is a very important, widely available biopolymer and polysaccharide of great industrial importance. It is obtained primarily from corn, potatoes, wheat, and rice [2,3,4,5]. Starch is often used in its natural form. It is a sugar composed of two polysaccharides, i.e., amylose and amylopectin. The ratio of amylose to amylopectin ranges from 1:3.5 to 1:5, and there are also starches that contain up to 98% of amylopectin, e.g., starch derived from corn, rice, and barley varieties [2,3,4,5,6,7]. What is important from a technological point of view is that amylose forms viscous solutions at 90 °C, while amylopectin forms a hydrogel with water at 70 °C. In addition, starch contains some amounts of non-saccharide components, such as protein (0.06–0.5%), fats (up to 0.9%), phosphorus (0.05–0.25%), and other minerals (including K, Na, Ca, Mg, Al, Fe). Starch also has very important biological functions in the human body. It is a source of energy for the central nervous system and red blood cells, and it is also necessary for the synthesis of amino acids and the oxidation of fatty acids in the body. In order to improve its functional properties or give it new features, it is mainly needed to decrease unfavorable properties, including high sensitivity to moisture and rather poor mechanical properties compared to synthetic polymers. Starch can be processed using traditional technologies, e.g., extrusion, embossing, and high-pressure injection. Modifications of native starch may also be helpful in increasing the functional properties [1,2,3,4,5,6,7,8,9,10,11,12]. 

The most commonly used physical modification is the gelatinization of starch accompanied by the destruction of the granular structure. During heating, most hydrogen bonds are broken, and a reduction in the melting point and glass transition is observable. Depending on the degree of destruction of the granular structure and the share of water, different products are obtained. On the other hand, chemical modifications may lead to a reduction in molecular weight, a disruption of glycosidic bonds in the chain, and consequently, a significant deterioration of the mechanical properties. The preparation of modified starches, mainly through esterification, allows them to obtain derivatives with the appropriate, expected hydrophobicity. However, a strategy based on chemical modification may be seriously limited by the toxicity, the high costs, and the removal of intermediate products. Therefore, scientists are looking for new, economical ways of modification to more biocompatible and multidirectional applications [8,9,10,11,12].

One of the applications for starch is its use as a substitute for fat and sugar. These types of substitutes perform numerous functions in food, e.g., they are fillers, stabilizers, and gelling and thickening agents, they affect the texture, maintain moisture, ensure proper crunchiness, and are also responsible for the taste sensation [5,6,7,8,9,10,11,12,13,14]. Texture is one of the most important features of food products. Thanks to the ability to bind water, starches that come from potatoes, waxy corn, and tapioca are used as thickeners in bakery products, fruit and vegetable products, meat products, frozen foods, and food concentrates. It is well known that starch properties are strictly dependent on the source of origin and the related gelatinization temperature. According to the literature, in the case of rice starch, the beginning and end of gelatinization are about 65 °C and 73 °C, respectively [11,12,13,14,15,16,17,18].

Another currently explored novel application of starch is the immobilization of enzymes, proteins, or drugs to improve catalytic activity or to create more efficient drug delivery systems [19]. On the other hand, S. Li and co-workers showed that the cross-linker-free chitosan/carboxymethylstarch–lysozyme microspheres could be a promising antibacterial additive, especially for *E. coli* for enteric infection treatment due to effectiveness and fast release at the intestinal tract. The development of an immobilized lysozyme is an urgent need in medicine, the food industry, and biotechnology. A higher antibacterial activity is possible owing to the synergistic effect between the membrane-attacking ability of chitosan and the cell wall-attacking ability of a lysozyme [8].

The aim of this manuscript was to characterize the combinations of starch with phospholipids and lysozymes (globular protein) to address two main issues: (a) enhancing starch’s functional properties or adding new features due to its influence on taste and texture and (b) exploring novel applications, like enzyme immobilization, for improved catalysis or drug delivery systems. Starch contains hydroxyl groups and simple glycosidic bonds that can be easily modified physically, chemically, or enzymatically to incorporate specific features, enhancing cooking properties, gel clarity, texture, adhesion, and film formation, increasing freeze–thaw stability, or reducing syneresis, retrogradation, and gelling tendencies [19]. Starch’s amphiphilicity, hydrophobicity, mechanical strength, and thermal stability are among the properties that can be improved as a result of starch modification. In the research described in this paper, surface hydrophobization was carried out using an *n*-alkane film without changing the bulk properties of starch, and it was checked how it would affect the properties of the entire system and the interactions between individual components. Starch modification usually leads to improvement of its mechanical properties and, above all, functional properties, and improves its processing parameters, which increases the possibilities of its use. 

When making modifications, it is important to add new quality features to the product and also preserve the structure-forming properties of native starch—thickening and gelling. Starch-based systems with phospholipids (e.g., phosphatidylcholine, PC main component of biological membranes that self-assemble at both hydrophilic and hydrophobic surfaces in aqueous media) can be used to obtain films suitable in pharmacy and medicine because their structure resembles cell membranes, making them biocompatible; they do not cause allergies and have a higher stability due to steric and electrostatic repulsions. Regarding phosphatidylcholine, PC is one of the main ingredients of the popular lecithin widely used in the food, cosmetics, and pharmaceutical industries [20]. Additionally, there may be a scope to obtain starch-based products (e.g., rice) with a lipid composition to reach the desired starch digestibility [21], whereas combinations with lysozymes, especially in the treatment of bacterial, fungal, and viral infections are useful [16,17,18,19]. A lysozyme is an antibacterial substrate, and proteins, glycoside hydrolase, and enzymes damage bacterial cell walls by catalyzing the hydrolysis of 1,4-beta-linkages between N-acetylmuramic acid and N-acetyl-D-glucosamine residues in a peptidoglycan and between N-acetyl-D-glucosamine residues in chitodextrins. A lysozyme is a part of the innate immune system and is a natural form of protection from Gram-positive pathogens, like *Bacillus* and *Streptococcus*. So, the incorporation of a lysozyme enhances the antimicrobial activity of starch-based systems. Modification by antibacterial substrate’s film may give new applications. Additionally, as was mentioned earlier, preparations of modified starch (e.g., acetylated annealed) may be deployed for a slow release of the therapeutic substances [22].

Regarding the above aspects, the physicochemical characterization of dispersion systems was aimed at determining the effect of starch modification and temperature of starch preparation, as well as checking the wettability parameters of starch-coated glass plates before and after modification [22,23,24,25]. Changes in effective diameter, multimodal size distribution, and electrokinetic potential (zeta) as a function of time were analyzed using the dynamic light scattering and microelectrophoresis technique, as well as the advancing and receding contact angle (and angle hysteresis), by the sessile drop method [26,27,28,29]. The obtained starch-based systems with strictly controlled parameters give the possibility of their multidirectional use [30,31,32,33,34,35,36]. 

Starch, because of its unique functional properties, is used in a number of novel foods, such as in the process of encapsulation of oils, aromas, and vitamins, as a pharmaceutical excipient, in a large number of agricultural products, and as a coating. However, such coatings are generally less permeable to gases and more permeable to water vapor due to the presence of significant amounts of hydrophilic groups, particularly hydroxyl groups. These properties can be modified by employing an appropriate procedure of biological alteration. In this type of product, water permeability is often not desired, hence the need to hydrophobize the surface. Hydrophobization using *n*-tetradecane film is one of the cheaper modification methods. These activities combined with modification with a lysozyme and phosphatidylcholine can lead to obtaining biodegradable starch with a wider range of applications without major costs. Starch is considered a biodegradable substance, and modifications with natural substances do not change this desirable property compared to other artificial polymers that do not usually have this feature [37,38,39,40,41,42,43,44,45,46]. Another scope of this manuscript was to find how the internal starch structure can influence its surface properties before and after biological modification. Such systems have been tested to obtain films applicable in food packaging or a suitable surface coating, e.g., pharmacy and medicine as an effect of potential biocompatibility with antiallergic, antibacterial, antifungal, and antiviral properties. To the best of our knowledge, these are the first reported studies of measuring advancing and receding contact angles on starch and modified starch films with additional hydrophobization and parallely studies of the physicochemical properties, including the stability of their analogous dispersions.

## 2. Results and Discussion

### 2.1. Effective Diameter and Zeta Potential of Starch-Based Systems vs. Time

During thermal technological processes, the stability of starch is improved by its cross-linking, especially in an acidic environment and during high shear. Unfortunately, such a modification also has negative effects, e.g., it reduces the transparency and clarity of gruels and resistance to cold storage [5,6,7,8,9,10,11,12,25,26,27,28,29]. Taking into account the gelatinization temperature for rice starch, three different temperatures were selected for testing: the temperature before the gelation point, the physiological temperature (useful for food and cosmetic products), and the temperature after the end of gelatinization.

**Figure 1:**
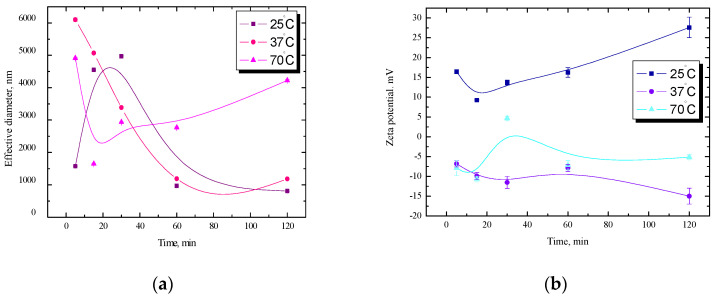
Dependence of the (**a**) effective diameter and (**b**) zeta potential of starch dispersion in a 10^−3^ M NaCl solution as a function of time.

In Figure 1, the relationships of the effective diameter and the zeta potential as a function of time for starch dispersion in a 10^−3^ M NaCl solution at three temperatures, 25 °C, 37 °C, and 70 °C, are presented. As was mentioned earlier, during gelatinization, firstly, the amorphous parts of the starch granule are hydrated, which induces swelling of the granules, leading to a distortion of the crystalline area. Secondly, the crystalline regions become more accessible to water. Thus, the size of starch granules increased with temperature increase from 25 °C to 70 °C (Figure 1a). 

The morphological changes associated with gelatinization are swelling and the rupture of the granular crystalline structure. At room temperature, a rapid increase in the effective diameter is visible within 30 min, while at 37 °C, the equilibrium state is reached faster, and the effective diameter values are more stable. After an hour at both temperatures, 25 °C and 37 °C, the diameters are noticeably more stable, and after 2 h, they are even similar. At 70 °C after 30 min, a monotonic increase in the diameter value was observed, which lasts up to 2 h of measurements and proves that the tested system at this temperature is the least stable. Analyzing the dependence of the zeta potential (Figure 1b), there are also clear differences between the samples, both in terms of the value of the potential and the nature of changes during the measurements. The obtained dependencies of the zeta potential as a function of temperature may be a further indication that starch gelatinization involves a change of the ordered state from the crystalline state to the disordered state. The zeta potential describes the forces occurring in the system and is indirectly a parameter of stability.

Starch granules are held together by hydrogen bonds and interchain hydrophobic bonds. As the temperature increases, the main process may be the breaking of hydrogen bonds between poly-(1 → 4)-glucan chains in crystallites and subsequent hydrophobic bonds. During these processes occurring in starch granules, after breaking hydrogen bonds, the polar groups probably hide inside and the hydrophobic parts remain outside; so as the temperature increases, a decrease in zeta potential is noticeable (Figure 1b). At temperatures of 37 °C and 70 °C, small negative zeta potential values were obtained, while at room temperature, these values are positive and the highest, which may indirectly confirm this conclusion and also indicate better stability. 

It is well known that the gelatinization process of starch is multi-stage, and its course strictly depends on the temperature. Typically, above the temperature of 50 °C, the hydrogen bonds between the amylose chains forming the crystalline fragments of the starch grain are broken. Water penetrates between them, creating new hydrogen bonds with released starch chains [18,19,20]. The gelatinization temperature is the temperature above which the starch does not show a granular structure in an aqueous medium and gives a paste characterized by high viscosity. Large grains lose their structure at the beginning. On the other hand, small grains are more resistant, which results in a large range of gelatinization temperature. This is why particle size measurements are so important in starch studies. 

As mentioned, the gelatinization temperature depends on the starch source and for rice starch, it is 65 degrees. Differences between rice varieties can be found in the ratio of amylose/amylopectin, internal ordering, and side chain length distribution. Rice starches have a better resistance to processing stress, are much more stable in time (very slow retrogradation), and have smooth gels with low structure. Starches with 25–30% amylose and 70–75% amylopectin are called “normal” starches; starches with very high levels of amylopectin (98–99%) are named “waxy” starches; and a third group contains starches with high amylose content (50–70%) and has no specific name [19]. The ratio of amylose to amylopectin in starch profoundly impacts its physicochemical properties or starch-based industrial products’ features, which, in turn, affect their functionality and potential applications. Higher amylose content implies increased film strength, while the presence of highly branched amylopectin leads to the formation of films with poor mechanical strength [19]. 

Remylgel belongs to waxy starches with a very high content of amylopectin. It is most probable that the branched chain of amylopectin is directed into the air in a gel film. Changing the parameters of starch during the gelification process (e.g., type of modification solution, temperature, time of modification), the wettability properties of the starch surface, and also the possibility of starch application, can be substantially changed. During gelatinization, the presence of a large amount of water is forming, and then the layered structure of the starch grains disappears and turns into bubbles. As a result of water entering the vesicles, amylose is completely dissolved, amylopectin swells more strongly, and peptization occurs [18,19,20,21,22,23,24,25]. These changes can also be seen in the example of measurements of size diameter and values of electrokinetic potential. For starch dispersion in a NaCl solution at 37 °C, slight fluctuations of this parameter were observed during 2 h of measurements. At 70 °C after 1 h, the zeta potential remained at the same level, about −7.0 mV. However, such a low potential does not guarantee the stability of the system; hence, large fluctuations of the effective diameter at this temperature are visible (Figure 1). At room temperature, higher potential values were noted, which resulted in more stable values of the effective diameter of starch dispersion in the NaCl solution. On this basis, a conclusion can be drawn about the correlation of these two parameters. 

**Figure 2:**
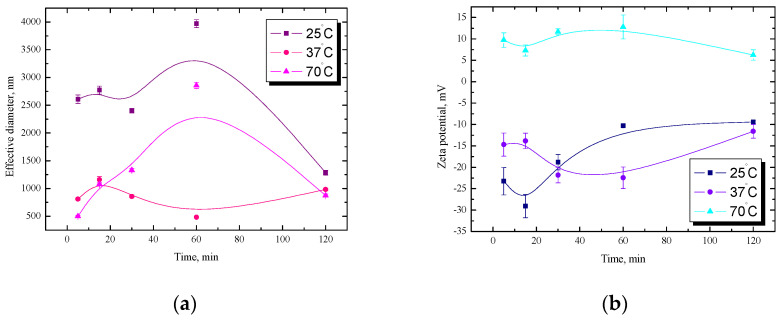
Dependence of the (**a**) effective diameter and (**b**) zeta potential of starch/phosphatidylcholine dispersion in a 10^−3^ M NaCl solution as a function of time.

Due to the potential medical and pharmaceutical applications, further studies were carried out for an analogous dispersion of starch with the addition of a phospholipid (PC). As already mentioned, the phospholipid increases the biocompatibility of such a system due to the fact that it is one of the main components of cell membranes. The obtained dependencies of the effective diameter and zeta potential as a function of time for the starch/phospholipid dispersions at three temperatures are presented in Figure 2. At room temperature and 70° during the first hour, large fluctuations are visible; however, after 1 h, in both cases, a monotonic decrease in the value of the effective diameter for up to 2 h of measurements is observable.

However, at a physiological temperature, the starch/PC dispersion in a 10^−3^ M NaCl solution is the most stable. The diameter values remained almost all the time at the same level of about 990 nm. Figure 2b shows the relationship of the zeta potential as a function of time. At room temperature and 37 °C, only negative values of the electrokinetic potential were recorded, unlike the measurements for the dispersion at 70 °C, which are only positive. This is directly related to the changes in starch structure below and above temperature gelatinization, as previously described in detail. The hydrophilic–hydrophobic character of starches depends on temperature, as well as their substitution type and starch pretreatment. At room temperature, the zeta potential values ranged from −29 mV to −9.4 mV, initially showing a decrease and then a monotonic increase. The reverse trend of changes was noted for the dispersion at 37 °C. The values of zeta potentials for room and physiological temperatures after 2 h are similar. On the other hand, the values of the electrokinetic potential of the starch/PC dispersion at 70 °C range from 6.2 mV to 9.7 mV, and fluctuations in time are visible. Such a low potential of approx. 10 mV does not guarantee the stability of the system; hence, large fluctuations of the effective diameter at this temperature are visible in Figure 2a.

**Figure 3:**
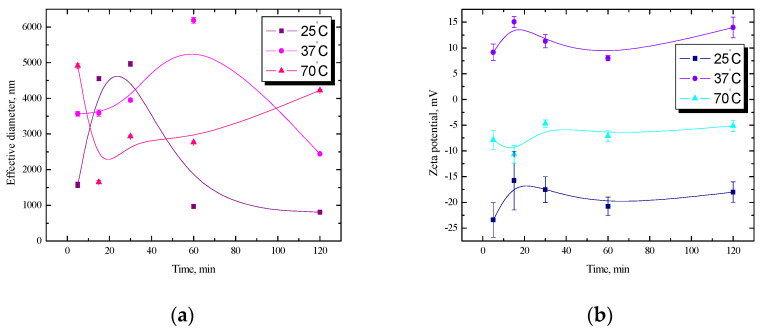
Dependence of the (**a**) effective diameter and (**b**) zeta potential of the starch/lysozyme dispersion in a 10^−3^ M NaCl solution as a function of time.

In the next stage of the research, measurements were carried out for the starch/lysozyme dispersion in a NaCl solution. The obtained effective diameter versus time relationships are shown in Figure 3a. The stability of dispersions depends on several factors, e.g., the type and concentration of the emulsifier used and its surface activity or adsorption rate at the interface. A lysozyme is a protein, and due to its surface properties, it can be used to create and stabilize different kinds of dispersions. Regarding emulsions, it is known that a protein film is formed around the oil droplets, which has a positive charge and prevents coalescence or flocculation. It has a high emulsifying capacity, especially after unfolding [15,16,17,18,19,20]. A rapid increase in the effective diameter is visible within 5–30 min and then a gradual decrease during 2 h at 25 °C. For the dispersion prepared at the physiological temperature (about 37 °C), nearly a double increase in diameter was observed during the first hour. The further course of the curve for 37 °C looks like a mirror image of the dependence obtained for the dispersion at 25 °C (an increase in the effective diameter was noted in the 60th minute, and then there was a decrease at 2 h in the opposite trend for physiological temperature).

At a temperature of 70 °C, a rapid decrease in the effective diameter is visible in the first 15 min, followed by a gradual increase. The obtained values of the zeta electrokinetic potential of the starch/lysozyme dispersion in the NaCl solution are shown in Figure 3b. It is easy to see that the course of the curves at 25 °C and 37 °C is very similar, but the electrokinetic potential values differ primarily in sign. The electrokinetic potential values at room temperature range from −23.4 mV to −18.0 mV. The positive charge from the protein film is noticeable at the physiological temperature. At this temperature, positive zeta potential values were obtained. This behavior may be related indirectly to the mechanism proposed lately [22]. The authors stated that the rearrangement of starch chains during heating, especially in the excess of water, leads to the strengthening of bonds between amylose and amylopectin, increasing starch granule stability, which can inhibit enzymatic hydrolysis. Moreover, this process can lead to the leaching of amylose chains from amorphous regions of starch granules, which probably reinforces their crystalline structure. The amorphous regions of semicrystalline starch granules represented regions most susceptible to the initial water absorption. The hydration of starch granules leads to increased mobility, especially in the amorphous regions. During progress in heating/hydration, the initially weaker or imperfect crystallites successively disappear, whereas the other ones become more perfect upon fusion and recrystallization.

**Figure 4:**
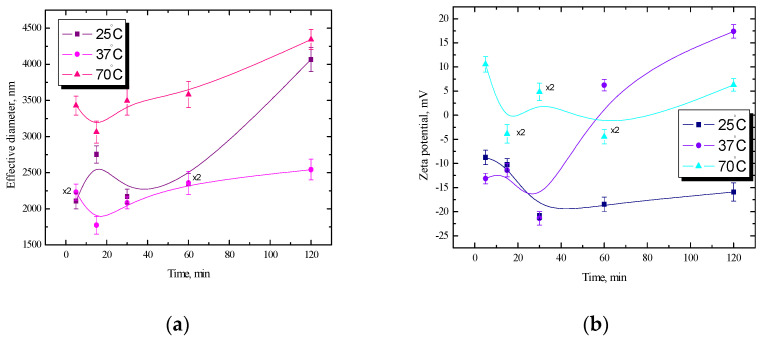
Dependence of the (**a**) effective diameter and (**b**) zeta potential of *n*-tetradecane/starch dispersion in a 10^−3^ M NaCl solution as a function of time.

At a temperature of 70 °C, within 15 min from the creation of the dispersion, a decrease in potential from −7.9 mV to −10.7 mV was noted, and then the potential remained at a constant level and reached a value of approx. −5.5 mV. Such a low potential does not guarantee the stability of the system; hence, large fluctuations of the effective diameter for this dispersion are visible in Figure 3a. In the case of the *n*-tetradecane/starch emulsion, fluctuations in the diameter value are visible at all temperatures, and the largest was at 37 °C; therefore, for better visibility, some results have been reduced twice (Figure 4a). On this basis, it can be concluded that the analogous starch dispersions are more stable than the tested *n*-tetradecane/starch emulsions. 

It is known that an O/W emulsion or its powdered form (dry emulsion) can be a carrier of oil-soluble compounds with health-promoting properties or biological activity (e.g., polyunsaturated fatty acids, sterols, vitamins, or antioxidants). A properly selected recipe and technology for the production of emulsion (dispersion) means that the structure of the particles must ensure the long-term stability of biologically active substances. For a high efficiency of such systems, it is necessary to disperse the oil phase to particles with a diameter of less than 1 mm using an appropriate homogenization technique, as the particle size also affects the stability of the emulsions produced [15,16,17,18,19,20]. 

The most important difference between proteins and other emulsifiers used is the size. Proteins having a high molecular weight diffuse into the interface much more slowly than a non-protein emulsifier. When the protein reaches the phase boundary, the chains unfold, and fragments are divided between the phases, which takes time. In the case of protein, to achieve the required value of interfacial tension to stabilize the emulsion, such a process may take up to several hours. Protein–polysaccharide combinations show very good stabilizing and emulsifying properties. This makes it possible to replace other low-molecular synthetic emulsifiers, which is advantageous for nutritional and pro-health reasons [30,31,32,33,34,35,36]. Therefore, the size will be particularly relevant for samples with lysozymes. In addition to the size and changes in the diameter of the tested systems, for a more complete characterization, the electrokinetic potential dependencies should also be considered. The most stable potential is visible at room temperature in the range from −20.8 mV to −18 mV. Despite quite stable values of the zeta potential, which usually guarantee the stability of the system (Figure 4b), the diameters are not stable (Figure 4a). This may be due to incomplete dispersion of the fat phase at this temperature. The homogeneity of the dispersion of the lipid phase should increase with increasing temperature. At a temperature of 37 °C, after the initial decrease in the zeta potential in 30 min to the value of −21.4 mV, an increase in the potential to the value of about 17.4 mV is visible. The greatest fluctuations of the potential over time were recorded at a temperature of 70 °C in a range from 10.6 mV to −4.5 mV, and the course of the curve resembles a sine wave (Figure 4b). 

It is well known that changes to temperature involved in food processing and products can also influence emulsion stability. For example, this type of research has been carried out for phospholipids, and the authors [24] have investigated the effect of the combination of starch and PC on the freeze–thaw stability of an O/W emulsion. The adsorption behavior, rheological properties, and mechanism of the improved stability of the starch–PC complex at the oil–water interface were discussed. The binding energy of the starch–PC complex was significantly lower than that of PC molecules, suggesting that the complex was relatively stable, and their changes were consistent with changes in electrostatic energy, indicating that such energy played a major role in the formation of the starch–PC complex. There were intermolecular hydrogen bonds and hydrophobic interactions in the starch–PC complex, while only hydrophobic interactions existed in the PC molecules [24].

### 2.2. Starch System without n-Tetradecane Film

**Figure 5:**
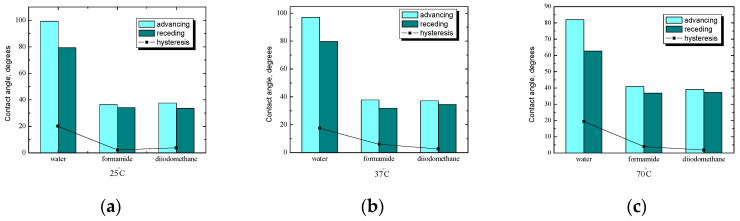
Comparison of the average value of the contact angle of the test liquid measured for the starch surface without *n*-tetradecane film. Starch preparation temperature (**a**) 25 °C; (**b**) 37 °C; (**c**) 70 °C.

One of the aspects of this research, apart from stability, was to investigate how the substrate and the modification of the starch layer will affect its wettability depending on the starch preparation temperature. The changes in bulk properties are usually seen in the surface properties of the starch. The parameters of wettability of the starch surface obtained also suggest topographical differences, as was concluded in our previous papers [26,27]. First, tests were carried out without *n*-tetradecane, and then, based on tests with *n*-tetradecane, the effect of starch hydrophobization was analyzed. Figure 5 shows the average values of the contact angle of starch plates without *n*-tetradecane film at three different temperatures. Liquids differing in polarity were used for wettability measurements, from highly polar water, through polar formamide, to non-polar diiodomethane. Natural starches have no affinity for hydrophobic substances, fats, or oils due to their hydrophilic nature. Specialized preparations consisting of two parts—hydrophilic and hydrophobic—are produced, having an affinity for both oil and water, so they can form stable oil-in-water emulsions. Starch with an additional layer can fulfill an analogous role, e.g., reduce the cholesterol content, and emulsifying substances make it possible to maintain a uniform structure in such products [15,16,17,18,19,20]. 

The methodology used for contact angle measurements also indirectly provides information about the changes in the structure of the starch layer before and after modification and the kind (or even strength) of forces between material molecules. The attractive forces between molecules that cannot be explained by ionic or covalent interactions can be caused by polar moments within molecules. During gelatinization, surface flatting can also be observed. Definitely, the highest average values of the contact angle at room temperature (Figure 5a) are noticeable for water. With the reduction in the polarity of the tested liquid, the hysteresis of the angle decreases because the differences between the values of the contact angles are small. For water, the hysteresis value is about 20°, which confirms the great sensitivity of starch to water. Starch absorbs water at any temperature, so even after a few seconds, its surface is completely different after placing a drop of water, while for the diiodomethane angle, hysteresis is only 2°. In the case of formamide and diiodomethane, the values of the contact angles are comparable.

It is well known that the contact angle depends on composition and temperature, as well as on the roughness of the film surface [18,19,20]. Determined by the rate of water drop absorption, starch films can be divided into two main groups (on the basis of contact angle values below 90° and over 90°). At the beginning of the experiment, starch surfaces prepared at 25 °C and 37 °C showed contact angles near 90°; after hydrophobization, contact angles were usually higher than 90°. Conversely, after PC or lysozyme modification, the contact angles were lower. The classification of starches according to the rate of water drop absorption gives temperature dependence. In our previous papers, the fastest rate of water drop absorption (the highest decrease in contact angle, about 0.1 degree/s) was observed for starch prepared at 70 °C after gelatinization [18,19,20]. A fast rate of water drop absorption was found for the water-soluble starches. Untreated starch at 70 °C showed a smaller contact angle than that at room and physiological temperatures. A reason might be the different retrogradation rates of the starch–water systems during film formation. Starch at room temperature before gelatinization shows the highest retrogradation rate of these untreated starches; therefore, the film is less hydrophilic and the contact angle increased [26,27]. 

A comparison of the average values of the contact angles of formamide and diiodomethane for starch-coated plates before hydrophobization is also shown in Figure 5. For formamide and diiodomethane, the angle values are very close to each other. The hysteresis value of the angle decreases with the decrease in the polarity of the tested liquids (the highest is for water and the lowest is for diiodomethane). Analyzing the above graphs, it can be concluded that the starch preparation temperature has practically no effect on the values of the contact angles of formamide and diiodomethane, while the influence is much greater for the contact angles of water. For the highest tested temperature, there was a decrease in the advancing angle of water by about 15 degrees. In addition, at each of the three temperatures, the angle hysteresis value decreases as the polarity of the liquid decreases.

### 2.3. Starch System with n-Tetradecane Film

**Figure 6:**
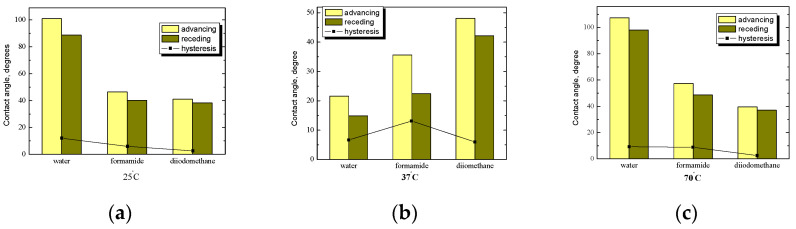
Comparison of the average value of the contact angle of the test liquid measured for the starch surface with *n*-tetradecane film. Starch preparation temperature (**a**) 25 °C; (**b**) 37 °C; (**c**) 70 °C.

For the systems with the addition of *n*-tetradecane, the effect of starch hydrophobization was analyzed. A comparison of the average values of the contact angle of water, formamide, and diiodomethane for starch-coated plates with *n*-tetradecane film is shown in Figure 6. At maximal temperature, both the average value of the contact angle and the hysteresis value depend on the polarity of the liquids used. The more polar the liquid is, the greater the contact angle and hysteresis. In the case of hysteresis, this is caused by differences between the values of the advancing and receding angles. At 70 °C, the hysteresis values of the water and formamide angles are very similar, approx. 10°, and for diiodomethane, they are half as much (about 5°).

As already mentioned, starch in food products also acts as a fat substitute. In low-fat products, when the degree of fat exchange exceeds 70%, the continuous phase of the product changes into an oil-in-water emulsion, changing the rheological properties and corresponding to condensed emulsions. The replacement of fat comes down to the introduction of water in combination with structure-forming additives in the form of hydrocolloids. The addition of starch has a significant impact on the mechanical and rheological properties of products, thanks to the ability of starch to create stable structures in the presence of water and under the influence of temperature. That is why physicochemical studies of oil/water emulsions with starch, as well as studies of the wettability of starch films, before and after hydrophobization are so important from the application point of view. At 37 °C, the lowest value of the contact angle was obtained for water and the highest for diiodomethane. On the other hand, regarding hysteresis, the highest value is obtained for formamide of approx. 13°, while water and diiodomethane were practically at the same level and amounted to approx. 7°. The chemical nature of the probe liquid, the size of its molecules, and the kind of interactions determine the surface penetration. The differences in the values of the contact angles and hysteresis manifest the strength of the solid/liquid interfacial interactions. 

Lately, to prove such a comment, the authors’ [47] independent gradient model was created to illustrate non-covalent interactions between starch and PC. For all studies of the the starch–PC complexes, the oil–water interfacial tension decreased quickly, indicating the adsorption of the complex onto the oil–water interface. There may be a formation of an interfacial layer in the starch–PC complex due to interactions between starch and PC, leading to a decrease in interfacial tension greater than that observed in the presence of single emulsifiers. Starch adsorbed slowly to oil surfaces due to its highly branched macromolecular structure and steric contribution and also formed a more extended organization at the interface and, as a result, a relatively thick layer. It is well known that in the case of water and formamide, the interactions are both dispersive and polar in nature, but for apolar diiodomethane, they are practically only dispersive. 

Decreasing contact angles of water indicates that during contact between water and the starch surface, its hydrophilicity increases. The largest hysteresis of water contact angles demonstrates that a water droplet penetrates the deepest into the layer structure and depends on surface preparation temperature. Emulsions prepared with the starch–PC complex had a relatively strong electrostatic repulsion that attenuated the oil droplets merging. No creaming and aggregates were observed in the emulsions prepared with starch–PC complexes, indicating that the low and moderate addition of starch helped stabilize PC-based emulsion during the freeze–thaw treatment. The improved stability may be related to the thickness and density of the interfacial layer formed by the complex. A number of studies supported the general idea that emulsifiers, which form a thick interfacial layer, are able to stabilize emulsions [47]. Comparing the average values of the contact angle of the tested liquids on the surface of starch-coated plates with and without the *n*-alkane film, it can be concluded that in this case, the effect of hydrophobization is also visible, mostly at physiological temperatures (Figure 6b).

### 2.4. Starch/Phosphatidylcholine Systems without n-Tetradecane Film

**Figure 7:**
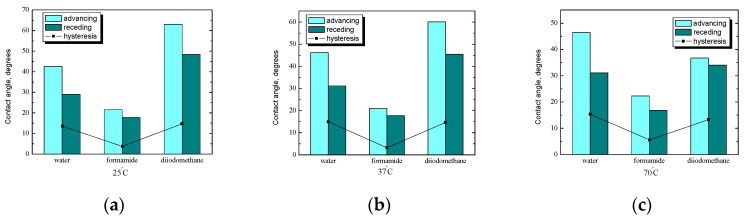
Comparison of the average value of the contact angle of the test liquid measured for the starch/PC surface without *n*-tetradecane film. Starch preparation temperature (**a**) 25 °C; (**b**) 37 °C; (**c**) 70 °C.

A comparison of the average values of the contact angles of water, formamide, and diiodomethane for starch/PC platelets without *n*-tetradecane film at three different temperatures is shown in Figure 7. As was mentioned earlier, phosphatidylcholine is used, e.g., in pharmaceutical technology due to its unique surface-active properties, and it acts as an emulsifier and a modifier of rheological properties, improving the stability and the consistency of dispersion. Phosphatidylcholine is non-toxic and biodegradable, so it can be used in parenteral nutrition preparations, such as dietary supplements, which lower the concentration of triglycerides and cholesterol in the blood. In addition to drugs, it can be used in the form of ointments, creams, emulsions, or suspensions. It is also important that as a result of combining starch with a phospholipid, the biocompatibility increases because these connections are similar in structure to biological membranes, and they do not cause sensitization or allergic reactions.

After PC treatment, the changes of advancing contact angles are visible, and the effect grows with the temperature increase. Both polar PC groups and patches of native starch are accessible for water during contact with liquid. Hence, the water contact angle can decrease. Due to the polar nature of water, its molecules exhibit a strong tendency to form hydrogen bonding. Therefore, the structural rearrangement of the PC molecules on the starch surface can occur, and the highest increase in the polarity of the starch surface after PC modification is observed. Wang and co-workers proved that the starch–PC complex at weight ratios of 3:7 and 5:5 remarkably improved the freeze–thaw stability of emulsions (no creaming process and creation of aggregates) and stated that it may be related to a thick and dense interfacial layer [24]. 

At room temperature (Figure 7a), the highest value of the contact angle was obtained for the non-polar liquid—diiodomethane and the lowest value was for formamide. In the case of non-polar diiodomethane, the value of the contact angle increased by about 20° compared to starch surfaces. The opposite situation is visible in the case of water, i.e., the average value of the angle is lower by more than half. The smallest value of the angle hysteresis was obtained for formamide, while for water and diiodomethane, the values were practically the same. The plates are definitely more hydrophilic compared to the basic ones. This confirms that a small modification can lead to obtaining systems with completely different properties, and the measurements of contact angles are very sensitive and, therefore, extremely useful parameters in determining such changes in a relatively simple and economical way. The hydrophilization of the starch surface after PC modification is caused by the rearrangement of the hydrophobic monolayer into layers with the polar groups of phospholipid heads exposed toward water drop. 

For starch/PC plates without *n*-tetradecane film (Figure 7b), at a starch preparation temperature of 37 °C, the graph looks analogous to that for room temperature (Figure 7a). A similar situation also occurs in the case of the highest temperature (Figure 7c), except that the highest value of the contact angle was recorded for water, and not, as in the case of two lower temperatures, for diiodomethane. After exceeding gelatinization, the temperature is completely different, and thus the phospholipid film on the starch surface shows a dissimilar character, which translates to the measured contact angles. The influence of PC is visible, especially for the most polar water and apolar diiodomethane, whose average values of the contact angle changed radically compared to the values of angles measured on plates covered with a layer of starch without modification. Other less sensitive methods would not show these changes, which, once again, confirm the high usefulness of wettability tests. Attractive interactions between starch and PC can cause strong complex formation and, as a result, film condensation and smoothing. Indeed, the low values of angle hysteresis may indicate surface smoothing. During temperature increases the homogeneity of the starch layer increases because the degree of gelatinization grows. An analogous conclusion was also drawn from the decreased values of the roughness parameters in our previous experiment for the same type of starch [26,27]. 

### 2.5. Starch/Phosphatidylcholine Systems with n-Tetradecane Film

**Figure 8:**
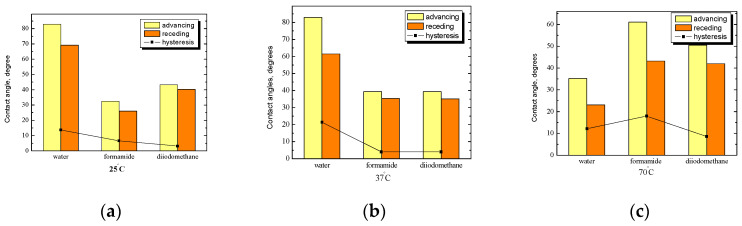
Comparison of the average value of the contact angle of the test liquid measured for the starch/PC surface with *n*-tetradecane film. Starch preparation temperature (**a**) 25 °C; (**b**) 37 °C; (**c**) 70 °C.

In Figure 8a, a comparison of the average values of the contact angle of water, formamide, and diiodomethane for starch/PC surfaces with additional *n*-tetradecane film is presented. At room temperature (Figure 8a), the highest value of the contact angle was obtained for water, and the lowest for formamide. As the polarity of the liquid used increases, the hysteresis of the angle also increases. In addition, as a result of hydrophobization, the value of the water contact angle increased by 40°, while for diiodomethane, the value of the contact angle decreased by approx. 20° (Figure 7 and Figure 8). The highest values of the contact angle and hysteresis were obtained for water, while the values of the formamide and diiodomethane angles and the hysteresis values for the contact angle of these two liquids were practically on the same level. The influence of PC on the structure of the starch layer on *n*-tetradecane film and its properties are very visible at a physiological temperature (Figure 8b), especially in the case of water, where the value of the contact angle increased almost four times (see Figure 6b).

At a temperature of 70 °C (Figure 8c), the trend of the measured contact angles and hysteresis changed significantly. This is the reverse of what is obtained at a physiological temperature. The value of the contact angle of formamide is the highest (at lower temperatures, the angle value was much lower), while in the case of water, the average value of the angle is lower, whereas at lower temperatures, it was much higher. 

### 2.6. Comparison of the Hysteresis Angle of Test Liquids for the Starch/Phosphatidylcholine System before and after Hydrophobization vs. Temperature

**Figure 9:**
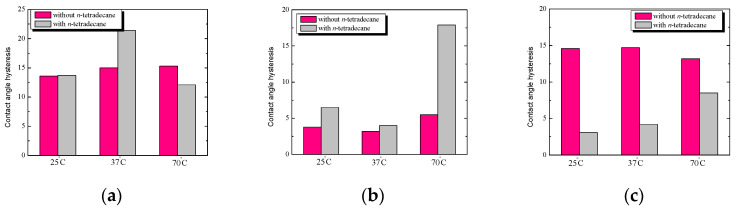
Comparison of the average value of the contact angle hysteresis for the starch/PC surface with and without *n*-tetradecane film for (**a**) water; (**b**) formamide; (**c**) diiodomethane.

In the next stage of this research, the hysteresis of water angles on the surface of starch with phosphatidylcholine (S/PC) with and without *n*-tetradecane film was compared, depending on the starch preparation temperature. At room temperature (Figure 9), the hysteresis values of the water angles for the S/PC plates without and with *n*-tetradecane film are practically the same and amount to about 13.5°. At a physiological temperature, the hysteresis value for the S/PC plate after hydrophobization is definitely higher (by several degrees) than before hydrophobization. 

The increase in attractive interactions between components causes the formation of more and more tightly packed monolayers characterized by lower values of the roughness parameters. Therefore, such condensed films are less permeable for the probe liquids used for the contact angle measurements. These suggestions have been observed in our previous research based on FTIR analyses [26,27]. Under the influence of modification with bioactive substances or by temperature increase, a change in the spectra of starch is possible. On the other hand, the authors [23] described that the inclusion complexes were formed by hydrophobic interactions between the alkyl chain of phosphatidylcholine and the debranched starch helix cavity. The complexes possessed a mass fractal structure, and a semicrystalline structure formed. After complexation, the stability of phosphatidylcholine was significantly improved. Additionally, this study revealed that debranched starch can be used as an effective carrier of phosphatidylcholine for the purpose of improving its stability [23].

At the highest temperature, the hysteresis value of the water angles for the tested plates without *n*-tetradecane film is higher than for the plates with the film. The obtained hysteresis values of the formamide angles for the starch/PC plates without and with *n*-tetradecane film, depending on the starch preparation temperature, are shown in Figure 9b. For PC, modified starch OH stretching and skeletal mode were slightly shifted compared to the native starch. Changes in the intensity of specific bands are found to be the major spectral ones during gelation. Gelatinization is primarily a hydration process, and chain conformation and helicity change when crystallinity and molecular orientation are lost. The signal around 1000 cm^−1^ is related to the intermolecular hydrogen bonding of hydroxyl groups or the plasticizing effect of water [26,27]. 

At each of the tested temperatures, the hysteresis values of contact angle for S/PC surfaces after hydrophobization are higher than before. The smallest difference in the angle values was obtained at the physiological temperature, while at 70 °C, the hysteresis value of the formamide angle for such surface with *n*-tetradecane film is as much as three times higher than for the same plate before hydrophobization. 

Completely different relations were obtained in the case of an apolar liquid (Figure 9c). The hysteresis values of the diiodomethane angles for S/PC plates without *n*-tetradecane film are much higher than for the same plates with the film; for example, at room temperature, the hysteresis values after hydrophobization (approx. 3°) are almost five times lower than before (approx. 15°). On this basis, it can be concluded that the change in starch temperature and the presence of *n*-tetradecane significantly affect the nature of the tested surface, and furthermore, a lower hysteresis value usually indicates smaller surface heterogeneity and, as a result better system reproducibility. Some other factors influence the surface parameters, such as the presence of unsaturated bonds in molecules and the phase state in which investigated films exist; therefore, FTIR spectra can be useful [26,27]. 

### 2.7. Starch/Lysozyme Systems without n-Tetradecane Film

**Figure 10:**
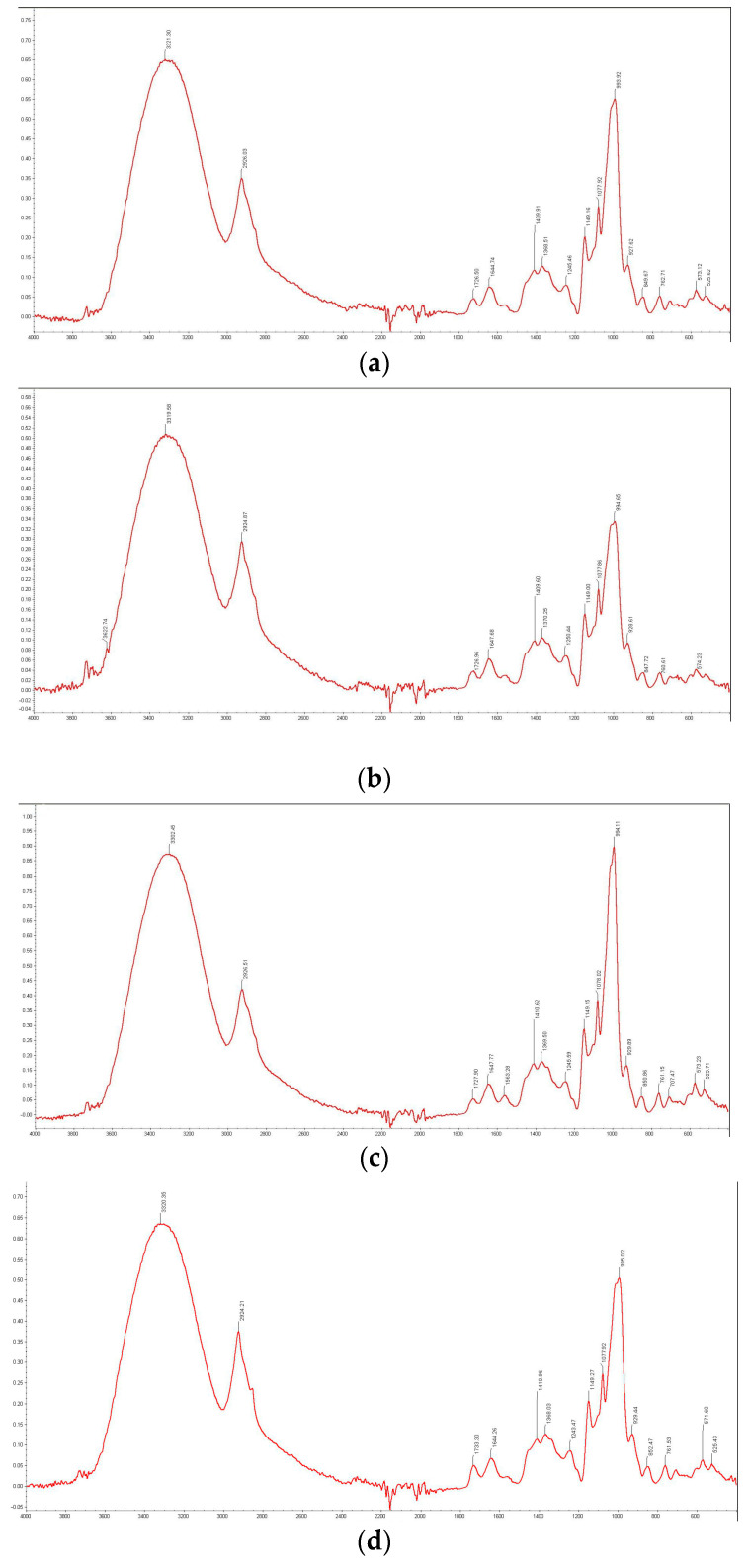

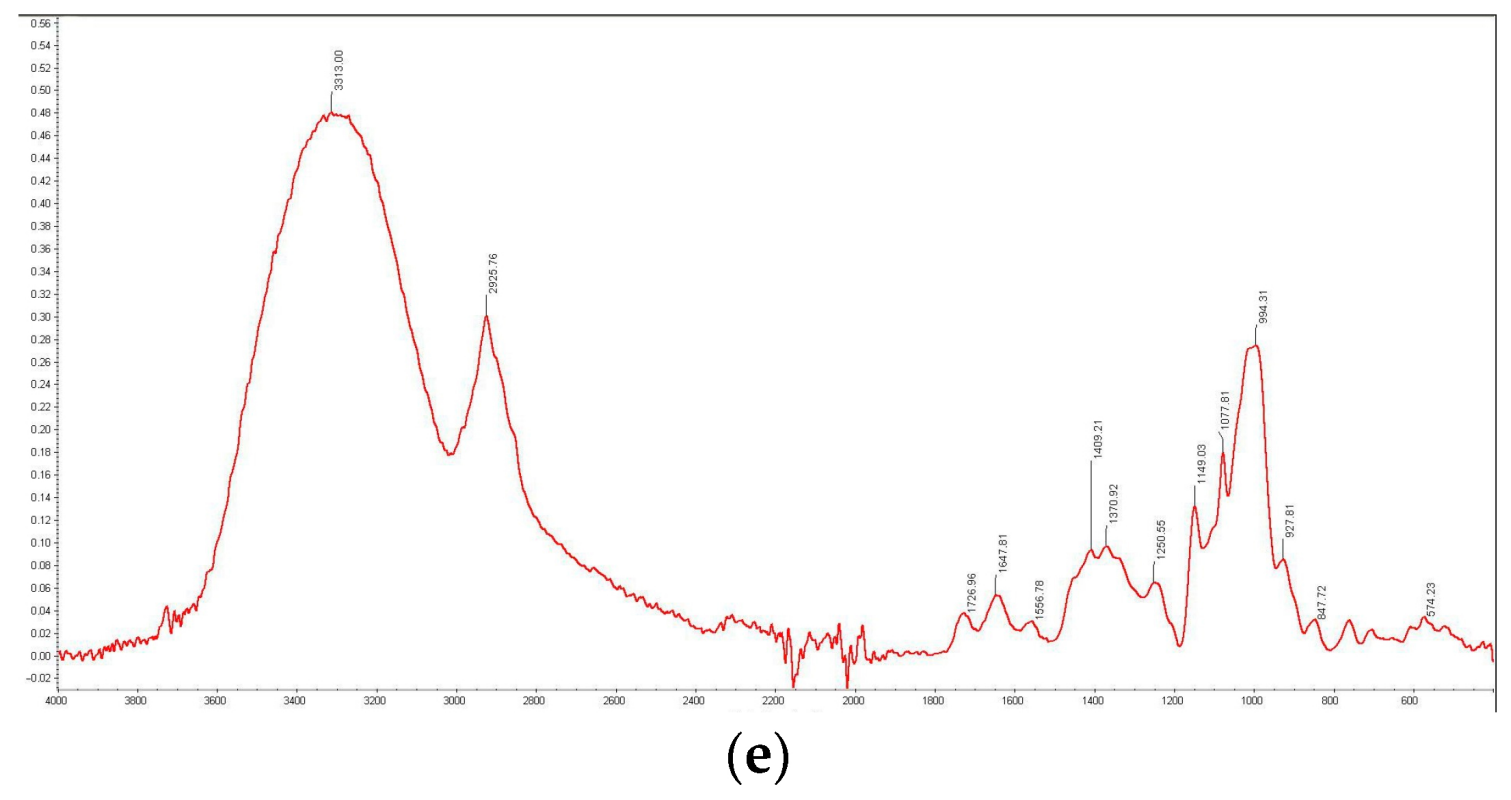
FTIR analysis for starches at different temperatures of (**a**) 25 °C, (**b**) 37 °C, (**c**) 70 °C, and after lysozyme modification, at (**d**) 25 °C, (**e**) 37 °C.

As was mentioned earlier, the condensed films are less permeable for the probe liquids used for the contact angle measurements. These suggestions can be observed as an effect of temperature. During gelatinization, the intensity of some bands increases, and new peaks can appear; hence, hydrophilic/hydrophobic changes can be observed from the FTIR spectra differences (Figure 10). During the gelatinization process, the most hydrophobic parts of the starch chain are directed into the air. However, steric limitations cause some polar groups to be present on the gel surface. The starch gel surface tends to reach maximal hydrophobic character with polar domains created by the functional glucose groups. It is most probable that the branched chain of amylopectin is directed into the air in a gel film. These are the primary results in the preparation of starch products of special properties, which can influence their solubility or polydispersity in aqueous media [47,48,49,50,51].

Starch is similar to other polysaccharides that absorb in the region 1200–800 cm^−1^. In this region, the average penetration depth is about 2 μm [26,27]. Because this value can be smaller than the average size of studied starch granules (1–3 μm), the acquired FTIR spectra are probably representative of only the external part of the starch granules. However, such spectra should be adequate for the starch film and compatible with the profilometer results. FTIR analysis of the untreated starch at three different temperatures is shown in Figure 10a–c. All spectra show that the absorption bands in the 900–1300 cm^−1^ region are sensitive to the gelation of starch, which mainly result from the CO and CC vibrational modes being highly coupled. As the absorptions in this region arise largely from C–O stretchings of the ring, linkages (C–O–C), and COH groups, the positions of these bands are similar in all carbohydrates. The native starch surface shows a typical pattern of Raman bands, i.e., skeletal vibrations below 800 cm^−1^, and the glycosidic linkage assigned to the band at about 940 cm^−1^. During the temperature increases, the breaking of hydrogen bonds between the chains in the crystallites and hydrophobic bonds may become a major event. Thus, the intensity of some bands changes (Figure 10b,c). The signal around 1000 cm^−1^ is related to the intermolecular hydrogen bonding of hydroxyl groups or the plasticizing effect of water. Absorbance at 1020 cm^−1^ is assigned to the vibration of C–O–H deformation and is related to changes in the amorphous and crystalline parts of the starch granule. The bands in the 1015–1025 cm^−1^ and 1040–1050 cm^−1^ regions are of particular interest because they are associated with the amorphous and crystalline structures of starch, and their ratio can estimate the extent of gelatinization [26,27]. 

The absorption bands at 1150, 1080, and 1017 cm^−1^ show a tendency to decrease in the bandwidth during gelation. Their narrowing can be interpreted as an evolution to a more uniform state of the amorphous phase (Figure 10a–c). The bands at 1080 cm^−1^ show the greatest shifts assigned to C–O and C–C stretching and C–C–O bending. During the process of starch gelatinization, the Raman bands are shifted to lower wavenumbers for the CH deformation and the skeletal and CH stretching modes, but for COH deformation, they shift to higher wavenumbers. At room temperature, the Lys-modified starches show differences compared to the native starch (Figure 10d), but at higher temperatures, the lysozyme effect is less visible (Figure 10e) because growing temperature affects the changes occurring during the gelatinization process in the system with and without lysozymes. This process is decisive, and biological additives affect the spectrum to a lesser extent. High temperature causes the quantitative denaturation of a native lysozyme within 20 min. The starch surface with the increasing temperature of preparation becomes more hydrophilic. The contact angles decrease with the temperature of starch preparation, which also increases the hydrophilic character of surfaces. In the case of water, the layer structure changes also result in large contact angle hysteresis. The wettability test together with FTIR analysis and optical profilometry are suitable and powerful methods to characterize the morphological and molecular properties of starch and modified starch. 

Accurate calculation of the area of the 1017–1022 cm^−1^ and 1044–1047 cm^−1^ regions and their ratios is commonly used in the literature [26,27] to estimate the extent of gelatinization. A lysozyme is an antibacterial substrate belonging to a large group of proteins and glycoside hydrolases. There are enzymes that damage bacterial cell walls by catalyzing the hydrolysis process of 1,4-beta-linkages between N-acetylmuramic acid and N-acetyl-D-glucosamine residues in a peptidoglycan and also between N-acetyl-D-glucosamine residues in chitodextrins. A lysozyme with these functions is recognized as part of the innate immune system, a natural form of protection against Gram-positive pathogens, like *Bacillus* and *Streptococcus* [16,17,18]. Therefore, covering starch films with lysozymes or their addition to dispersion systems will result in additional desirable properties both in the food and pharmaceutical industries. On the other hand, the immobilization of lysozymes on solid supports has lately shown positive results, as demonstrated by the increased stability and extended half-life of the enzyme in the research by Anastas and co-workers [5]. Furthermore, the immobilization of the lysozyme improves the ability of the lysozyme to target not only Gram-positive bacteria but also Gram-negative bacteria. The large effect of the lysozyme is noticeable, similar to the case of starch modification with a phospholipid, at 37 °C (Figure 10e). This may be related to conformational changes that occur in the protein structure on the surface of partially gelatinized starch. 

**Figure 11:**
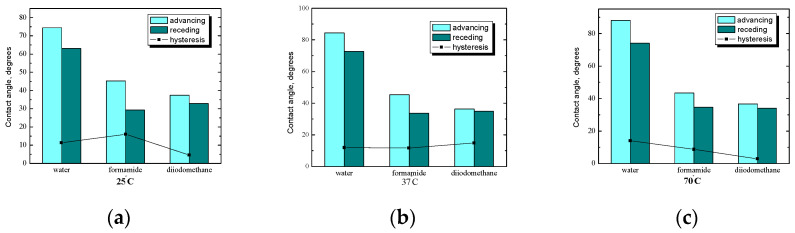
Comparison of the average value of the contact angle of the test liquid measured for the starch/lysozyme surface without *n*-tetradecane film (starch preparation temperature (**a**) 25 °C; (**b**) 37 °C; (**c**) 70 °C).

A comparison of the average values of the contact angle of water, formamide, and diiodomethane for plates coated with starch and a lysozyme without *n*-tetradecane film is shown in Figure 11. At room temperature, the value of the contact angle increases with increasing polarity of the liquid used; the highest values were obtained for water, and the lowest for diiodomethane. However, for hysteresis, the relationship was not so clear, and the values obtained are as follows. For water, about 10°, for formamide, as much as 17°, and for diiodomethane, only 6°. Applying an additional layer (lysozyme) to the starch-coated plates increased the contact angle values by several degrees in the case of formamide and diiodomethane, while the average value of the water contact angle decreased by several degrees, which may indirectly indicate an increase in surface hydrophilicity.

At a physiological temperature (Figure 11b), there is an analogy to measurements at room temperature (Figure 11a). It is easy to see that the values of the contact angles change in a similar way, i.e., the more polar the liquid is, the higher the values of the contact angles (Figure 11a). Additionally, the hysteresis value for polar liquids is practically at the same level of about 12°, while for non-polar diiodomethane, it is larger and amounts to about 16°. At a temperature of 70 °C (Figure 11c), the average values of the contact angles did not change compared to the measurements at lower temperatures (Figure 11a,b). Noticeable changes are seen in the hysteresis values, which decrease as the polarity of the liquid used decreases. The greatest difference between the advancing and receding angles is for water, and the smallest is for diiodomethane. The starch preparation temperature did not affect the contact angle values in these systems, while the application of protein on the starch increased the contact angles.

### 2.8. Starch/Lysozyme Systems with n-Tetradecane Film

**Figure 12:**
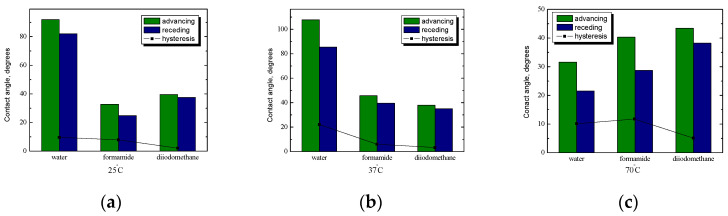
Comparison of the average value of the contact angle of the test liquid measured for the starch/lysozyme surface with *n*-tetradecane film (starch preparation temperature (**a**) 25 °C; (**b**) 37 °C; (**c**) 70 °C).

The graph (Figure 12) shows the average values of the contact angles together with the hysteresis values of water, formamide, and diiodomethane for S/Lys plates with *n*-tetradecane film. When the starch preparation temperature was 25 °C (Figure 12a), the highest value of the contact angles was obtained for water, and the lowest for formamide. As in most cases, the angle hysteresis varies depending on the polarity of the liquid used. The less polar the liquid, the smaller the hysteresis of the angle. In the case of physiological temperature (Figure 12b), both the value of the angle hysteresis and the average values of the contact angle increase with the increase in the polarity of the liquid used. For diiodomethane, which is non-polar, the lowest values were obtained, while for polar water, the values of the contact angle and hysteresis were the highest. 

The values of the contact angles obtained for the S/Lys plates with *n*-tetradecane film (starch preparation temperature of 70 °C) are shown in Figure 12c. Both the value of the advancing and receding angle increases as the polarity of the liquid used decreases. The hysteresis values for water and formamide are very similar. A significantly lower hysteresis value was noted for diiodomethane. The application of an additional layer on a plate with starch-coated *n*-tetradecane film caused an increase in the contact angles of polar water and non-polar diiodomethane.

### 2.9. Comparison of the Hysteresis Angle of Test Liquids for the Starch/Lysozyme System before and after Hydrophobization vs. Temperature

**Figure 13:**
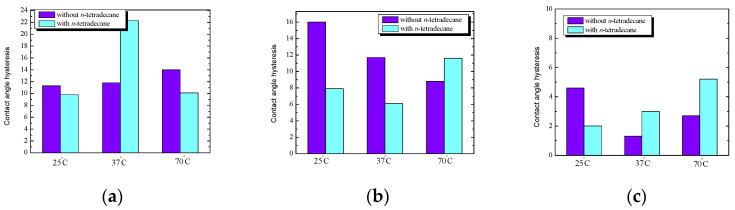
Comparison of the average value of the contact angle hysteresis for the starch/lysozyme surface with and without *n*-tetradecane film for (**a**) water; (**b**) formamide; (**c**) diiodomethane.

In Figure 13, the hysteresis of water, formamide, and diiodomethane angles on the S/Lys surface with and without *n*-tetradecane film was compared depending on the starch preparation temperature. In Figure 13, at 25 °C and 70 °C, the hysteresis values of the water contact angle for S/Lys plates with *n*-tetradecane film are very similar and amount to about 10°. At physiological temperature, the effect of *n*-tetradecane is most noticeable. For room and physiological temperature, the hysteresis values are twice as high for the S/Lys plates without *n*-alkane film compared to the same plates with the film.

At a temperature of 70 °C, the effect of hydrophobization is more visible; the hysteresis value before hydrophobization is 9°, and after hydrophobization, it is 12°. In addition, it can also be seen that the hysteresis values for the S/Lys plates without *n*-tetradecane film decrease with increasing temperature. This can be explained by the easier spreading of the starch solution at higher temperatures and the concomitant decreasing film thickness. The hysteresis of diiodomethane angles of the starch/lysozyme system before and after hydrophobization is shown in Figure 13c, and these are the smallest hysteresis values that we obtained. As the temperature increases, the hysteresis value for the S/Lys plates with *n*-tetradecane film increases, i.e., the effect of hydrophobization also increases. This can be explained by the fact that large surface changes occur under the influence of *n*-tetradecane and temperature, which are visible on the basis of contact angle measurements. As the temperature increases, the breaking of hydrogen bonds between the poly-(1 → 4)-glucan chains in the crystallites and hydrophobic bonds may become a major event. The loss of crystallinity may be considered a manifestation of starch gelatinization.

**Figure 14:**
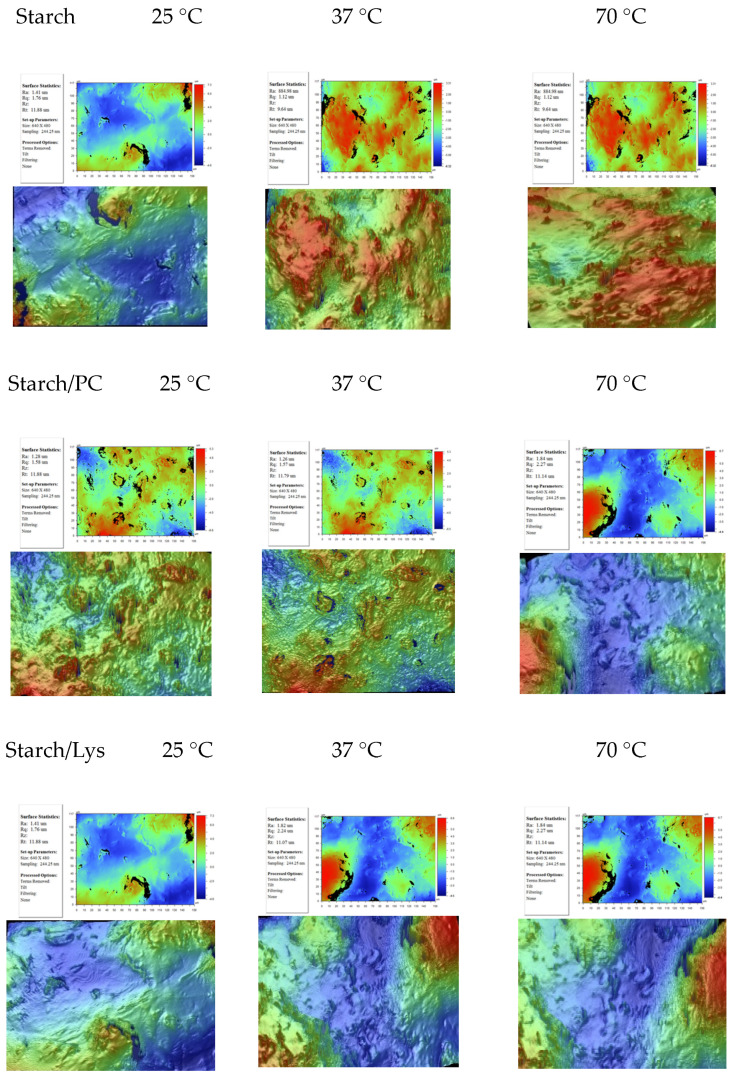
Images obtained from an optical profilometer for starch before and after modification at different preparation temperatures.

Another currently explored novel application of starch is the immobilization of enzymes, proteins, or drugs to improve the catalytic activity of enzymes used in food technology or to create more efficient drug delivery systems [19]. The research carried out strictly fits into this trend of potential applications. A detailed description of topographic changes was presented in our previous works [26,27]. The roughness and topography of the starch surface were investigated using an optical profilometer (Contour GT-K1, Veeco) measuring topography with high accuracy from a subnanometer up to 10 mm in size. We have performed such measurements in three different sites for glass plates covered with native starch and its modification. Below, we present some sample surfaces (Figure 14). 

The obtained images of the starch surface by profilometer show topographical differences between the native starch surface and that modified by a biological solution. For example, the height distribution shows that the starch surface is covered mostly by patches. The hydrophilization of the starch surface after PC modification is caused by the rearrangement of the hydrophobic monolayer into bilayer patches with the head polar groups exposed toward water drop. Attractive interactions between starch and PC may cause complex formation. This strong association can cause film condensation and smoothing. Indeed, the monolayer of PC on the starch makes the surface smoother. Reduced values of all roughness parameters were obtained compared to the native starch surface; although, in order to explain changes in surface roughness, the type and size of interactions between starch molecules and the additional layer should be taken into account. Due to the polar nature of water, its molecules have a strong tendency to form hydrogen bonds. Therefore, there may be a structural rearrangement of PC or Lys molecules on the starch surface and an increase in the polarity of the starch surface after its modification. This effect decreases as the temperature increases. On the other hand, the lysozyme reduces the hydrophobic nature of the starch surface.

In both cases, during temperature increase, the homogeneity of the starch layer increases because the degree of gelatinization grows. Moreover, one can also find some correlations between the changes in surface roughness and the values of contact angles measured on native and modified starch surfaces. The increase in attractive interactions between both components causes the formation of more and more tightly packed monolayers characterized by lower values of the roughness parameters. Therefore, such condensed films are less permeable for the probe liquids used for the contact angle measurements. The chemical nature of the probe liquid, the size of its molecules, and the kind of interactions determine the surface penetration. It is clear that the differences in the values of the contact angles and hysteresis manifest the strength of the solid/liquid interfacial interactions. It is obviously known that the contact angle measured on such surfaces is of macroscopic quantity, which is, in fact, an averaged (apparent) value of the microscopic contact angles. The reproducibility of the contact angle values and the topography images enable full surface characterization.

### 2.10. Starch Surface Wettability: Work of Adhesion

The changes in wettability and the hydrophilic/hydrophobic character of the starch surface before and after modification vs. temperature can be also depicted by calculating the work of probe liquids adhesion. Details of this procedure were presented in our previous papers [26,27]. In the case of polar liquids (water or formamide), the changes in the values of work of adhesion indicate changes in polar interactions. However, if the work of adhesion and correlated surface energy is calculated from apolar diiodomethane contact angles, the values characterize the London dispersion interactions on the surface and usually are the most stable. This indirectly informs us what impacts we are dealing with in a specific case. The changes in the work of adhesion calculated from water angles are much greater than in the case of diiodomethane. In all the presented systems (Table 1), both the hydrophobization effect and the influence of temperature are noticeable.

Also, the temperature effect is more noticeable. For starch, as a function of temperature, an opposite trend of changes in the adhesion work was observed, depending on the substrate, increasing without *n*-tetradecane and decreasing after hydrophobization. For S/PC and S/Lys substrates, such a clear trend of changes is difficult to capture. On the other hand, the effect of the hydrophobic substrate is less visible in the values of the adhesion work calculated on the basis of diiodomethane contact angles. This is understandable due to the type of interactions in this case, and values in terms of 16–18 mJ/m^2^ are often repeated.

## 3. Materials and Methods

### 3.1. Materials

The following reagents were used in the research without further purification: *n*-tetradecane (≥99% GC, Fluka, Steinheim, Germany), rice starch for food purposes, that is naturally sourced, sustainably produced from renewable feedstocks, and GMO free (not genetically modified, Remylgel 663 DR-P, HORTIMEX, Wielsbeke, Belgium), L-α-phosphatidylcholine from egg yolks, p.a. (SIGMA-Aldrich, Steinheim, Germany), lysozymes (SIGMA, St. Louis, MO, USA), NaCl (p.a. POCH S.A. Gliwice, Poland), pentane (≥99% GC, Fluka), diiodomethane (p.a. POCH S.A. Gliwice, Poland), formamide (p.a. POCH S.A. Gliwice, Poland), water from the Millipore Q system, and a resistivity 18.2 MΩ cm. Products marked as GMO free contain, consist of, or have been produced from organisms/raw materials for which there are equivalents entered in the community register of genetically modified foods, and these organisms do not contain, consist of, or have not been produced from GMOs.

#### 3.1.1. Solution Preparation

A total of 1 g of starch (1 mg of lecithin or 0.25 mg of lysozyme) was weighed into a 50 mL volumetric flask and supplemented to the mark with 10^−3^ M NaCl solution. Depending on the purity of the ingredients used, the proportions of the molecular weight of lysozyme to lecithin range from 5 to 15; the lowest proportion of 1:5 was chosen in the research. A solution of *n*-tetradecane in pentane was made as follows: 10 mL of pentane was poured into a 25 mL beaker and 0.1 mL of *n*-tetradecane was pipetted. The solution prepared in this way was poured with a pipette onto a glass plate (optical glass plates, As Polonia, Warsaw, Poland). In the case of hydrophobized plates, after one day, the pentane evaporated, leaving only *n*-tetradecane on the surface.

#### 3.1.2. Preparation of Glass Plates

Optical glass plates were used for the measurements. The solution of *n*-tetradecane prepared for wettability tests, described in Section 3.1.1, was poured with a pipette onto plates; next, the pentane was evaporated. The other solutions were poured onto plates or plates previously coated with a solution of *n*-tetradecane in pentane. Glass plates were used as solid substrata for covering with starch solution at three different temperatures (25 °C, 37 °C, and 70 °C). After reaching the sample preparation temperature, the appropriate volume of the native starch (partially gelatinized or gelatinized) solution was slowly spread on glass plates to obtain a homogeneous layer. Next, the plates were kept at room temperature for drying, and after that, they were placed in desiccators filled with silica gel for about 1 day to perform experiments. 

#### 3.1.3. Emulsion Preparation

To prepare the *n*-tetradecane/starch emulsion in NaCl solution, 1 mg of starch was weighed into a 100 mL volumetric flask, and 0.1 cm^3^ of *n*-tetradecane was pipetted and filled up to the mark with a 10^−3^ M NaCl solution. It was homogenized for 15 min at 10,000 rpm in a homogenizer (SilentCrusher M, Heidolph, Schwabach, Germany). After homogenization, the emulsion was placed in measuring cuvettes, and measurements were carried out as a function of time. Measurements of analogous systems were made at 25 °C (room temperature), 37 °C (physiological), and 70 °C (after starch gelatinization).

### 3.2. Methods

During the experimental part, a laboratory scale (Sartorius, Göttingen, Germany), a homogenizer, a zetameter (ZetaPlus, Brookhaven, Holtsville, NY, USA), and a contact angle measurement apparatus (GBX, Rhone, France) were used.

#### 3.2.1. Dynamic Light Scattering

The phenomenon of dynamic light scattering is used to measure the particle size of dispersed systems from a few nm to a maximum of 1 μm. This method is based on measuring changes in a specific light wavelength (in this type of system of 670 nm) and fluctuations in average intensity. The radiation scattered by the particles is registered with a detector placed at a certain angle to the incident beam. The intensity of the scattered radiation fluctuates constantly as the dispersed particles undergo continuous Brownian motion and thermal motion. When the particles are small, they exhibit fast diffusion motions and, therefore, fast fluctuations. When larger particles are present in the sample, they diffuse more slowly and thus cause slower fluctuations. 

Two physical processes, i.e., particle size increase due to coalescence or flocculation and particle migration, which leads to creaming or sedimentation, often result in system instability. Detection of these processes at an early stage and kinetic analysis of changes in these destabilizing processes is very important in predicting stability. One of the ways to indirectly determine the stability of the dispersion system is the analysis of changes in particle diameter and zeta potential. The computer coupled with the device (Zeta Plus/Pals) automatically controls the course of the correlation function, including the selection of the sampling time and the measurement time. Using the method of dynamic light scattering, assuming that non-interacting spherical particles are dispersed in a medium with a viscosity *η*, the diffusion coefficient with the particle size *d*(*H*) can be related by the Stokes–Einstein equation (Equation (1)) [33] as follows:

(1) $$D=\frac{kT}{3\pi \eta }d(H)$$

where *k* is the Boltzmann constant, *T* is the absolute temperature, *η* is the medium viscosity, and *d*(*H*) is the particle size.

Importantly, for the same portion of the sample, both the particle size and the electrokinetic potential were determined using an apparatus (zetameter) in thermostatic polyacrylic cuvettes as a function of time, i.e., after 5 min, 15 min, 30 min, 1 h, and 2 h from the moment of emulsion (dispersion) preparation, depending on the temperature, i.e., at 25 °C, 37 °C, and 70 °C. 

#### 3.2.2. Microelectrophoresis

Electrophoresis is one of the known methods for determining electrokinetic potential. It consists of the movement of charged colloidal particles relative to the solvent under the influence of the applied electric field of constant intensity *ε.* These particles move with a characteristic speed, which depends on the field strength, the viscosity of the medium, and the zeta potential. According to Henry’s theory, the electrophoretic mobility of particles of any size can be expressed by the following formula (Equation (2)) [33,34,35,36]:

(2) $$U_{e}=\frac{\varepsilon \zeta }{\eta }f(\kappa a)$$

where *U_e_* is electrophoretic mobility, *ε* is permittivity, *ζ* is electrokinetic potential, *η* is medium viscosity, and *f*(*ĸa*) is the Henry function (*a* is particle radius, *κ* is the Debay—Hückel parameter, depending on electrolyte concentration, *1/κ* is diffusion layer thickness).

For small values of *ĸa* (*ĸa* ≤ 1), the Henry function takes the form of the Hückel equation (Equation (3)) as follows:

(3) $$U_{e}=\frac{2\varepsilon \zeta }{3\eta }$$

For large values of *ĸa*, the Smoluchowski equation is used (Equation (4)).

(4) $$U_{e}=\frac{\varepsilon \zeta }{\eta }$$

The zeta potential can be determined for the same batch of emulsion (dispersion) for which the mean or effective diameter was measured, which is an advantage of the instrument used. After measuring the effective diameter, an electrode is placed in the measuring cuvette and electrophoretic mobility is obtained by microelectrophoresis. The computer then converts the measured mobility into electrokinetic potential according to the Smoluchowski or Hückel equation (Equations (3) and (4)) [34,35,36,37]. 

#### 3.2.3. Contact Angle Measurements

Measurements of contact angles were carried out using the GBX apparatus by the sessile drop method using liquids that differed in polarity, i.e., strongly polar water, polar formamide, and non-polar diiodomethane. The determination of the hydrophilic/hydrophobic character of the starch surface before and after modification can be used in the food and pharmaceutical industries [26,27,28,29]. Switchable wettability may be a convenient parameter providing information about even subtle changes of starch surface properties. Additionally, modification, for example, by an antibacterial substrate’s film, may give new applications of starch. During these experiments on the surface of glass plates coated with starch, such as *n*-tetradecane (or both) or starch modified by phospholipids or lysozymes, a drop of the test liquid with a volume of 3 µL was placed, and the advancing contact angle was measured. Then, 1/3 of the liquid volume was taken from the drop, and the receding contact angle was measured. Hysteresis was defined as the difference between these two angles. For each system, 10 to 20 contact angle measurements were made, depending on the type of liquid used. For various types of modifications, measurements of both angles and determining the hysteresis provided appropriate results and the possibility of in-depth interpretation.

## 4. Conclusions and Future Perspective

The studies of dispersion systems with the participation of starch as an emulsifier and additional bioactive substances are current and interesting due to the possibility of their versatile use in the pharmaceutical, medicine, and food/packaging industries. In this aspect, it is also very helpful to determine the hydrophilic/hydrophobic nature of the starch surface. For all systems, changes in zeta potential were usually correlated with changes in the effective diameter. Moreover, the starch dispersions were more stable than analogous *n*-alkane/starch emulsions at room and physiological temperatures. With regard to the combination of starch and lysozyme, it was found that the stability of the *n*-tetradecane/starch Lys system in the electrolyte solution increases with increasing temperature; the most stable one was obtained at 70 °C. Increasing the starch preparation temperature and the use of a lysozyme film contributed to an increase in the average contact angle values.

In the case of the tested *n*-alkane/starch and the PC system, the increase in temperature did not enhance its stability; however, for plates coated with unmodified starch and starch/PC without the *n*-alkane film, the contact angle hysteresis values decreased with a temperature increase, pointing to a more homogeneous surface. The highest wettability was obtained for the starch/PC both with and without the *n*-tetradecane film, where the values of water angles at each of the tested temperatures are the smallest, in the range of 35°–80°, which means moderately or softly hydrophilic character, optimal for biological samples. The effect of hydrophobization was visible for each of the tested liquids and temperatures, decreasing vs. the thickness of the substrate increase. Starch applied to the plate with the *n*-alkane film forms a less uniform layer than that directly applied to glass. The most homogeneous surfaces were acquired for pure starch at 70 °C; the hysteresis angle values decrease with increasing temperature, which can be explained by the fact that starch increases the degree of swelling with increasing temperature and spreads better on glass. However, after starch modification, the highest hysteresis values were recorded for the S/PC and S/Lys plates at physiological temperatures, and the effect of hydrophobization was most visible at this temperature.

To generalize, the obtained results proved that switchable wettability may be a convenient parameter providing information on surface properties. The values of contact angles are apparent but in connection with hysteresis, they provide an opportunity to determine the energetic state of the starch surface via the starch/probe liquid interfacial interactions. The contact angles change with the temperature of starch preparation and different types of modification, which means that the hydrophilic/hydrophobic character of surfaces can be moderated. The wettability test together with stability measurements are suitable and powerful methods to characterize the strength and specificity of interactions that describe many biological processes, whereas a combination of bioactive substances and different treatment parameters led to the improvement of biopolymer wettability and functionality, and the incorporation of additional features into the final product without significantly changing the beneficial properties of native starch. We hope that these results will be helpful for new applications of such starch-based polysaccharide systems. 

## Figures and Tables

**Table 1:** Work of adhesion [mJ/m^2^] calculated from water (W) or diiodomethane (D) contact angles for the starch surface before and after modification vs. preparation temperature.

Temp./ Surface	Without *n*-Tetradecane			With *n*-Tetradecane		
	S	S/PC	S/Lys	S	S/PC	S/Lys
25 °C W	75.70	43.68	85.3	135.58	129.35	0.41
37 °C W	82.95	41.34	141.94	102.51	141.94	30.79
70 °C W	129.34	0.56	145.55	100.14	11.02	139.39
25 °C D	64.35	70.71	64.35	2.92	30.48	16.92
37 °C D	16.92	2.42	99.32	18.28	16.92	16.92
70 °C D	16.92	89.62	99.32	16.92	88.50	78.99

## Data Availability

The data will be shared by the authors.
